# Effects of dietary incorporation of linseed oil with soybean isoflavone on fatty acid profiles and lipid metabolism-related gene expression in breast muscle of chickens

**DOI:** 10.1017/S1751731120001020

**Published:** 2020-11

**Authors:** Z. Y. Gou, X. Y. Cui, L. Li, Q. L. Fan, X. J. Lin, Y. B. Wang, Z. Y. Jiang, S. Q. Jiang

**Affiliations:** Institute of Animal Science, Guangdong Academy of Agricultural Sciences, State Key Laboratory of Livestock and Poultry Breeding, Key Laboratory of Animal Nutrition and Feed Science in South China, Ministry of Agriculture and Rural Affairs, Guangdong Key Laboratory of Animal Breeding and Nutrition, Guangzhou 510640, China

**Keywords:** growth performance, n-3 polyunsaturated fatty acid, meat quality, antioxidation, health indicator

## Abstract

The meat quality of chicken is an important factor affecting the consumer’s health. It was hypothesized that n-3 polyunsaturated fatty acid (n-3 **PUFA**) could be effectively deposited in chicken, by incorporating antioxidation of soybean isoflavone (**SI**), which led to improved quality of chicken meat for good health of human beings. Effects of partial or complete dietary substitution of lard (**LA**) with linseed oil (**LO**), with or without SI on growth performance, biochemical indicators, meat quality, fatty acid profiles, lipid-related health indicators and gene expression of breast muscle were examined in chickens. A total of 900 males were fed a corn–soybean meal diet supplemented with 4% LA, 2% LA + 2% LO and 4% LO and the latter two including 30 mg SI/kg (2% LA + 2% LO + SI and 4% LO + SI) from 29 to 66 days of age; each of the five dietary treatments included six replicates of 30 birds. Compared with the 4% LA diet, dietary 4% LO significantly increased the feed efficiency and had no negative effect on objective indices related to meat quality; LO significantly decreased plasma triglycerides and total cholesterol (**TCH**); abdominal fat percentage was significantly decreased in birds fed the 4% LO and 4% LO + SI diets. Chickens with LO diets resulted in higher contents of *α*-linolenic acid **(**C18:3n-3), EPA (C20:5n-3) and total n-3 PUFA, together with a lower content of palmitic acid (C16:0), lignoceric acid (C24:0), saturated fatty acids and n-6:n-3 ratio in breast muscle compared to 4% LA diet (*P* < 0.05); they also significantly decreased atherogenic index, thrombogenic index and increased the hypocholesterolemic to hypercholesterolemic ratio. Adding SI to the LO diets enhanced the contents of EPA and DHA (C22:6n-3), plasma total superoxide dismutase, reduced glutathione (**GSH**)/oxidized glutathione and muscle GSH content, while decreased plasma total triglyceride and TCH and malondialdehyde content in plasma and breast muscle compared to its absence (*P* < 0.05). Expression in breast muscle of fatty acid desaturase 1 (***FADS**1*), *FADS2*, elongase 2 (***ELOVL**2*) and *ELOVL5* genes were significantly higher with the LO diets including SI than with the 4% LA diet. Significant interactions existed between LO level and inclusion of SI on EPA and TCH contents. These findings indicate that diet supplemented with LO combined with SI is an effective alternative when optimizing the nutritional value of chicken meat for human consumers.

## Implications

Dietary linseed oil can increase the content of n-3 polyunsaturated fatty acid in chicken meat, this effect being potentially increased with soybean isoflavone which has strong antioxidant activity. Indeed, dietary linseed oil improves tissue fatty acid profiles and, when distributed with SI, increases the proportion of long chain n-3 polyunsaturated fatty acid in meat and levels of indicators of antioxidant status in muscle and plasma of chicken. These dietary strategies are effective methods to improve the nutritional value of chicken meat.

## Introduction

Chicken is a major source of meat, which is popular with consumers all over the world. The nutritional quality of chicken meat is a significant factor affecting the consumer’s health. The n-3 polyunsaturated fatty acid (**PUFA**) in human nutrition have drawn considerable interest due to their potential health benefits, such as contributing to the development of infant brain and decrease in the risk of tumors, cardiovascular diseases, and inflammatory disorders (Ruxton *et al.*, 2007; Riediger *et al.*, 2009). The most significant n-3 PUFAs are *α*-linolenic acid (**ALA**, C18:3n-3), EPA (C20:5n-3) and DHA (C22:6n-3). Importantly, ALA serves as a precursor of other n-3 long-chain fatty acids such as EPA and DHA (Rymer and Givens, 2005). The nutritional profile of meat products can be improved by replacing saturated fatty acids (**SFAs**) with n-3 PUFA in animal diets. Linseed oil (**LO**) is the most commonly used nutritional lipid source for its high content of ALA, and supplementation of the chicken diet with LO can increase the content of n-3 PUFA in chicken meat products (Kartikasari *et al.*, 2012). The potential for unsaturated fatty acids, especially the n-3 PUFA, to undergo deleterious oxidation can be offset by simultaneous inclusion of antioxidants in the diet (Kouba and Mourot, 2011). Soybean isoflavones (**SIs**), as phenolic compounds including genistein, daidzein and glycitein, are the main phytoestrogens of soybeans (Setchell, 1998). The SIs have strong antioxidant activity *in vivo* and *in vitro* (Liu *et al.*, 2005; Jiang *et al.*, 2007a) and their dietary inclusion improve growth performance and meat quality by decreasing lipid peroxidation and improving antioxidative status in broiler chickens (Jiang *et al.*, 2007b; Jiang *et al.*, 2014).

In the present study, the lard (**LA**)-containing basal diet of chickens was modified by substituting half or all of the LA with LO, with or without an effective quantity of SI. The objective was to assess the effects of such dietary regimens on the growth performance, biochemical indicators, meat quality, fatty acid profiles, lipid-related health indicators and expression of genes related to lipid metabolism in the breast muscle of chickens.

## Material and methods

### Birds, diets and experimental design

A total of 900 1-day-old fast-growing Chinese yellow-feathered male chicks (Lingnan, an improved meat-type breed with good meat quality that is popular locally) were obtained from Guangdong Wiz Agricultural Science and Technology Co. Ltd. (Guangzhou, China). All birds were fed a standard starter diet from 1 to 29 days of age. They were weighed at day 29 (BW and SEM 597 ± 2.00 g) and randomly allotted to a total of 30 floor pens (1.3 m × 3.5 m) in an environmentally controlled room for the 37-day experiment during the grower and finisher phases (day 29 to 66). Pens were then randomly assigned to the five dietary treatments, each of which included six replicates of 30 birds. The control group was fed a basal diet containing 4% LA and the four treatment diets received the basal diet modified as follows: 2% LA + 2% LO; 4% LO; or the latter two diets containing 30 mg SI/kg (2% LA + 2% LO + SI and 4% LO + SI). The SI was from Newlands Feed Science and Technology, Guangzhou, China. Experimental diets were formulated based on the Chinese Feeding Standard of Chicken published in 2004 and the Feed Database in China in 2018. The first three diets are described in Table 1, and the SI was added to the premix used for the last two. The fatty acid composition of diets is presented in Table 2. Feed and water for consumption were provided *ad libitum*. Mortality of birds was recorded daily. At day 66 (typical market age), the birds were deprived of feed for 12 h and reweighed to calculate the average daily gain (**ADG**). Average daily feed intake (**ADFI**) was recorded on a replicate basis and the feed/gain ratios were calculated.

**Table 1:** Composition and nutrient levels of experimental diets of yellow-feathered chickens from 29 to 66 days of age (% as fed basis)

Components	29 to 49 days			50 to 66 days		
	4% LA	2% LA + 2% LO (with or without SI)	4% LO (with or without SI)	4% LA	2% LA + 2% LO (with or without SI)	4% LO (with or without SI)
Ingredient (%)						
Corn	61.8	61.8	61.8	65.7	65.7	65.7
Soybean meal	26.3	26.3	26.3	19.1	19.1	19.1
Corn gluten meal	3.0	3.0	3.0	4.0	4.0	4.0
Lard	4.0	2.0	0	4.0	2.0	0
Linseed oil	0	2.0	4.0	0	2.0	4.0
l-Lysine HCl	0.13	0.13	0.13	0.14	0.14	0.14
dl-Methionine	0.11	0.11	0.11	0.13	0.13	0.13
Limestone	1.11	1.11	1.11	1.12	1.12	1.12
Dicalcium phosphate	1.73	1.73	1.73	1.58	1.58	1.58
Salt	0.26	0.26	0.26	0.26	0.26	0.26
Zeolite powder	0.56	0.56	0.56	2.97	2.97	2.97
Premix^1^	1.00	1.00	1.00	1.00	1.00	1.00
Total	100.00	100.00	100.00	100.00	100.00	100.00
Nutrient composition^2^						
Metabolizable energy (MJ/kg)	13.09	13.04	12.98	13.10	13.04	12.99
CP (%)	19.02	19.02	19.02	17.02	17.02	17.02
Crude fat (%)	6.89	6.89	6.89	6.87	6.87	6.87
Lysine (%)	1.00	1.00	1.00	0.85	0.85	0.85
Methionine (%)	0.41	0.41	0.41	0.41	0.41	0.41
Methionine + cysteine (%)	0.71	0.71	0.71	0.69	0.69	0.69
Calcium (%)	0.90	0.90	0.90	0.85	0.85	0.85
Non-phytate phosphorus (%)	0.40	0.40	0.40	0.37	0.37	0.37

LA = lard; LO = linseed oil; SI = 30 mg/kg soybean isoflavone.

1 Provided the following per kilogram of diet in two feeding stages, Vitamin B_1_, 2.4, 2.1 mg; Vitamin B_2_, 4, 3 mg; niacin, 17, 15 mg and choline chloride, 600, 500 mg, separately; Vitamin A, 6000 IU; Vitamin D_3_, 500 IU; Vitamin E, 20 IU; Vitamin K_3_, 0.50 mg; pantothenic acid, 10 mg; Vitamin B_6_, 3.5 mg; biotin, 0.15 mg; folic acid, 0.55, mg; Vitamin B_12_, 0.01 mg; Fe, 80 mg; Cu, 7 mg; Mn, 60 mg; Zn, 75 mg; I, 0.35 mg; Se, 0.23 mg.

2 Values were calculated from data provided by Feed Database in China (2018).

**Table 2:** Fatty acid composition in the experimental diets of yellow-feathered chickens from 29 to 66 days of age

Fatty acid (%)	29 to 49 days			50 to 66 days		
	4% LA	2% LA + 2% LO	4% LO	4% LA	2% LA + 2% LO	4% LO
C14 : 0	0.83	0.46	0.12	0.82	0.47	0.13
C16 : 0	18.55	13.52	8.64	18.51	13.52	8.65
C16 : 1n-7	1.64	0.88	0.14	1.63	0.87	0.14
C18 : 0	8.71	5.89	3.15	8.61	5.81	3.09
C18 : 1n-9	34.83	27.80	20.95	34.90	27.89	21.04
C18 : 2n-6	28.50	29.77	31.01	28.88	30.09	31.18
C18 : 3n-3	1.42	16.54	31.24	1.30	16.25	30.67
C20 : 0	0.91	0.90	0.76	0.95	0.91	0.84
C20 : 1n-9	0.81	0.79	0.77	0.83	0.77	0.81
C20 : 4n-6	0.98	0.48	0.05	0.97	0.48	0.07
C22 : 0	0.98	0.99	0.94	1.01	0.96	0.95
C24 : 0	0.58	0.55	0.42	0.57	0.52	0.49
MUFA	37.29	29.47	21.86	37.37	29.54	21.99
PUFA	30.89	46.79	62.30	31.14	46.82	61.93
SFA	30.55	22.30	14.04	30.47	22.19	14.17

LA = lard; LO = linseed oil; MUFA = monounsaturated fatty acid; PUFA = polyunsaturated fatty acids; SFA = saturated fatty acid.

### Sample collection

Birds were sampled on day 66. Following the 12-h overnight fast, 12 chickens per treatment (two birds per replicate) with BW close to the replicate average were chosen. Heparinized blood samples were collected from the brachial vein; plasma samples were separated by centrifugation of blood at 1200×**g** for 10 min at 4°C and plasma aliquots were kept at −80°C. The birds were then electrically stunned and exsanguinated before obtaining the tissues. The dressed weight was recorded; breast muscle was quickly sampled from the same area on the right side, snap frozen in liquid nitrogen and stored at −80°C for RNA extraction and biochemical analysis. The remaining breast muscle from the right side was used to measure the intramuscular fat (**IMF**) content and fatty acid analysis, and the left breast muscle was used for the determination of meat quality indicators. Abdominal fat was removed and weighed, and the abdominal fat percentage was calculated (abdominal fat weight as a percentage of dressed weight).

### Biochemical analysis

Frozen muscle tissues of 40 mg in 4 ml of homogenization buffer (0.05 M Tris-HCl, pH 7.4, 1 mM EDTA, 0.25 M sucrose) were homogenized on ice with an Ultra-Turrax (T8; IKA-Labortechnik, Staufen, Germany) for 5 s at 13 500 rpm. The homogenate was centrifuged at 12 000×**g** for 10 min at 4°C and the supernatant was stored at −80°C. The activity of total superoxide dismutase (**T-SOD**) and the contents of total antioxidant capacity (**T-AOC**), reduced glutathione (**GSH**), oxidized glutathione (**GSSG**) and malondialdehyde (**MDA**) were determined using colorimetric methods to measure thiobarbituric acid reacting substances with a spectrophotometer (Biomate 5; Thermo Electron Corporation, Rochester, NY, USA). The assays were conducted with the kits purchased from Nanjing Jiancheng Institute of Bioengineering (Nanjing, Jiangsu, China) following the manufacturer’s procedures. The GSH/GSSG ratio was subsequently calculated. The plasma concentrations of total triglyceride (**TG**), total cholesterol (**TCH**), high-density lipoprotein cholesterol (**HDL-C**) and low-density lipoprotein cholesterol (**LDL-C**) were measured by an autoanalyzer (Selectra ProXL Clinical Chemistry System, the Netherlands) using commercial kits (Biosino Bio-technology and Science Inc., Beijing, China).

### Meat quality measurements

Samples of left pectoral muscle were used to measure the color, pH, drip loss and shear force. Meat color was measured at 45 min postmortem using a Chroma Meter to measure CIE LAB values (L*, relative lightness; a*, relative redness, b*, relative yellowness). The pH was measured at 24 h postmortem in the *pectoralis major* muscle with a portable pH meter equipped with an insertion glass electrode. The drip loss of breast muscle was estimated using the method described by Rasmussen and Andersson (1996); samples were placed in a plastic bag filled with air and fastened to avoid evaporation and held at 4°C for 24 h, and the drip loss was determined by weighing. The shear force was determined using an Instron Universal Mechanical Machine (Instron model 4411; Instron Corp., Canton, MA, USA) as follows: after storage for 24 h at 4°C, the samples were heated until the internal temperature was 75°C. Samples (1.0 cm × 1.0 cm × 2.5 cm) were taken from each meat sample and sheared in triplicate perpendicular to the fiber orientation. The IMF content in the breast muscle was determined by extraction with petroleum ether in a Soxhlet apparatus. IMF is expressed as a percentage of the dry weight of the muscle.

### Fatty acid composition and analyses of lipid-related health indicators

In preparation for the analysis of fatty acid composition, lipids from samples were extracted with diethyl ether/petroleum ether (1 : 1, vol : vol) after being hydrolyzed. Fatty acid methyl esters (**FAMEs**) of total lipids were prepared for gas chromatographic determination by the Standard Determination of Fatty Acids in food in China (GB5009.168-2016). An Agilent 6890 gas chromatography system (Santa Clara, CA, USA) fitted with a TR-FAME chromatographic column (100.0 m × 250 µm × 0.20 µm) was used for separating and quantifying the FAME with internal standard (C11:0). The composition of the fatty acids is expressed as percentages of total fatty acids.

The lipid-related health indicators of breast muscle were estimated by the n-6:n-3 ratio and the following three indicators: atherogenic index (**AI**), thrombogenic index (**TI**) and hypocholesterolemic/hypercholesterolemic ratio (**h** : **H**). The AI and TI were calculated according to Ulbricht and Southgate (1991); AI = [C12:0 + (4 × C14:0) + C16:0]/[(PUFA) + (monosaturated fatty acid, **MUFA**)], and TI = [C14:0 + C16:0 + C18:0]/[(0.5 × MUFA) + (0.5 × n-6) + (3 × n-3) + (n-3/n-6)]. The h : H was calculated according to Fernández et al. (2007); h : H = (C18 : 1n-9 + C18 : 1n-7 + C18 : 2n-6 + C18 : 3n-6 + C18 : 3n-3 + C20 : 3n-6 + C20 : 4n-6 + C20 : 5n-3 + C22 : 4n-6 + C22 : 5n-3 + C22 : 6n-3)/(C14 : 0 + C16 : 0).

### RNA extraction and real-time quantitative PCR

Total RNA was isolated from 100 mg of frozen breast muscle using an extraction kit (Invitrogen, Carlsbad, CA, USA), according to the manufacturer’s protocol. The concentration and purity of RNA were determined using a NanoDrop ND-1000 spectrophotometer (NanoDrop Technologies, Wilmington, DE, USA). The OD260/280 values of all samples were limited to the range of 1.8 to 2.0. The RNA samples were reverse transcribed using M-MLV reverse transcriptase (Promega, Madison, WI, USA), and the specific primers (Supplementary Table S1) of nine genes were designed using Primer Premier 6.0 software, including fatty acid desaturase 1 (***FADS**1*), *FADS2*, fatty acid elongase 2 (***ELOVL**2*), *ELOVL5*, fatty acid synthase (***FAS***), lipoprotein lipase (***LPL***), 3-hydroxy-3-methylglutaryl-coenzyme A reductase (***HMGCR***), carnitine palmitoyltransferase-1α (***CPT**1α*) and sterol regulatory element binding protein-1 (***SREBP**-1*) genes.

The complementary DNA (**cDNA**) was amplified by quantitative PCR with a CFX96 Real-time System (Bio-Rad, Hercules, CA, USA) using an iTaq™ Universal SYBER^®^ Green Supermix (Takara Biotechnology, Dalian, China). After initial denaturation for 3 min at 95°C, amplification was performed for 39 cycles (95°C for 15 s, 30 s at the annealing temperature and 72°C for 30 s). The specificity of the PCR products was evaluated by the analysis of melting curves. The comparative CT method was used to determine the fold changes in gene expression, with the fold change calculated as 2^−ΔΔCT^. The results are expressed as the mean fold change in gene expression from triplicate analyses, using control group samples as the calibrators (arbitrarily assigned an expression level of 1 for each gene). Negative controls, without a cDNA template, were included in this experiment.

### Statistical analysis

Replicate served as the experimental unit. All five diets were assessed using one-way ANOVA and, where appropriate, means were compared by Tukey tests. Values are reported as means ± SEM derived from the error mean square for *n* = 6. The few significant interactions between the level of LO substitution and addition of SI were examined by two-way GLM in SAS (version 9.1; SAS Institute, Cary, NC, USA), with exclusion of the basal diet; the remaining diets constitute a 2 × 2 factorial design. Differences were considered to be statistically significant at *P* < 0.05.

## Results

### Growth performance

Compared to the 4% LA diet, substitution of half or all of the LA with LO significantly decreased the ADFI of chickens (2% or 4% LO *v*. 4% LA); the final BW and ADG of chickens from 29 to 66 days were not affected (Table 3).

**Table 3:** Effects of dietary linseed oil supplementation, with and without soybean isoflavone, on growth performance of yellow-feathered chickens from 29 to 66 days of age^1^

Variable	4% LA	2% LA + 2% LO	4% LO	2% LA + 2% LO + SI	4% LO + SI	SEM	*P* values^2^			
							Treatment	LO	SI	LO × SI
Final BW (g)	1828	1804	1838	1804	1801	6.530	0.27	0.27	0.19	0.20
ADFI (g)	92.07^a^	88.23^b^	88.82^b^	88.18^b^	87.58^b^	0.467	0.010	0.99	0.41	0.45
ADG (g)	32.40	31.77	32.65	31.77	31.67	0.180	0.31	0.31	0.21	0.21
Feed/gain (g/g)	2.85^a^	2.78^ab^	2.72^b^	2.78^ab^	2.77^ab^	0.013	0.045	0.20	0.47	0.36
Survival rate (%)	98.34	98.89	98.89	97.22	98.89	0.383	0.61	0.58	0.78	0.46

LA = lard; LO = linseed oil; SI = 30 mg/kg soybean isoflavone; BW = body weight; ADG = average daily gain; ADFI = average daily feed intake.

1 Data are means for *n* = 6 replicates (30 birds/replicate).

2 Treatment, all five diets. LO, dietary linseed oil substituted for half or total LA in the last four diets. SI, dietary LO with or without SI in the last four diets.

a,b Means marked with different letters in rows differ significantly at *P* < 0.05.

### Indicators of lipid metabolism and antioxidation

As shown in Table 4, there were no significant (*P* > 0.05) differences in the antioxidant index in plasma and breast muscle when half or all of the LA were substituted with LO in chickens (2% LA + 2% LO or 4% LO *v*. 4% LA). The plasma content of T-SOD in birds given 4% LO with SI was higher (*P* < 0.05) than in birds given LO without SI. There were no significant effects of the five diets on the T-AOC, GSH and GSSG contents of plasma, but the GSH/GSSG ratio was increased when the SI was added to diets containing LO (2% LA + 2% LO + SI or 4% LO + SI *v*. 2% LA + 2% LO or 4% LO; *P* < 0.05). Adding SI to the diets containing LO significantly increased GSH content and GSH/GSSG ratio in breast muscle and decreased the concentration of MDA in plasma and breast muscle compared to its absence.

**Table 4:** Effects of dietary linseed oil supplementation, with and without soybean isoflavone, on lipid metabolism and antioxidation indicators of yellow-feathered chickens^1^

Variable	4% LA	2% LA + 2% LO	4% LO	2% LA + 2% LO + SI	4% LO + SI	SEM	*P* values^2^			
							Treatment	LO	SI	LO × SI
Plasma										
TG (mmol/l)	0.802^a^	0.622^b^	0.490^c^	0.496^c^	0.379^d^	0.029	0.001	0.001	0.001	0.73
TCH (mmol/l)	2.73^a^	2.56^ab^	2.38^b^	2.30^b^	1.66^c^	0.079	0.001	0.001	0.001	0.023
HDL-C (mmol/l)	2.10	2.00	2.04	2.06	1.89	0.028	0.16	0.23	0.48	0.077
LDL-C (mmol/l)	0.444	0.405	0.422	0.360	0.389	0.016	0.55	0.55	0.87	0.54
T-AOC (U/ml)	6.43	7.13	7.07	7.80	7.17	0.181	0.22	0.38	0.36	0.50
T-SOD (U/ml)	265.11^ab^	258.19^b^	242.63^b^	276.97^ab^	296.80^a^	5.939	0.038	0.85	0.004	0.13
GSH (mg/l)	9.13	9.16	8.78	10.03	9.36	0.181	0.28	0.19	0.077	0.71
GSSG (μmol/l)	12.57	13.13	11.80	10.89	10.34	0.405	0.16	0.17	0.012	0.57
GSH/GSSG	0.770^ab^	0.713^b^	0.747^b^	0.922^a^	0.912^a^	0.028	0.027	0.75	0.001	0.55
MDA (nmol/ml)	2.48^a^	2.46^a^	2.34^a^	1.93^b^	2.01^b^	0.038	0.045	0.83	0.012	0.83
Breast muscle										
GSH (mg/g prot)	5.50^ab^	5.12^b^	5.02^b^	7.65^a^	6.78^a^	0.101	0.047	0.50	0.010	0.58
GSSG (μmol/l)	17.21	17.42	17.33	16.67	17.02	0.398	0.089	0.27	0.065	0.67
GSH/GSSG	0.322^c^	0.295^c^	0.290^c^	0.459^a^	0.403^b^	0.0112	0.034	0.15	0.001	0.23
MDA (nmol/mg prot)	1.34^ab^	1.41^a^	1.42^a^	1.10^b^	1.24^ab^	0.021	0.034	0.23	0.022	0.43
Abdominal fat (%)	2.88^a^	2.67^ab^	2.15^b^	2.39^ab^	2.23^b^	0.086	0.024	0.042	0.76	0.23

LA = lard; LO = linseed oil; SI = 30 mg/kg soybean isoflavone; T-AOC = total antioxidant capacity; T-SOD = total superoxide dismutase; GSH = reduced glutathione; GSSG = oxidized glutathione; MDA = malondialdehyde; TG = total triglyceride; TCH = total cholesterol; HDL-C = high-density lipoprotein cholesterol; LDL-C = low-density lipoprotein cholesterol.

1 Data are means for *n* = 6 replicates (2 birds/replicate).

2 Treatment, all five diets. LO, dietary LO substituted for half or total LA in the last four diets. SI, dietary linseed oil with or without SI in the last four diets.

a,b,c,d Means marked with different letters in rows differ significantly at *P* < 0.05 (*n* = 6).

Compared with the chickens fed the 4% LA diet, the contents of TG and TCH decreased by dietary inclusion of LO, and especially in the presence of SI, where the levels of TG and TCH were further decreased (*P* < 0.05), as shown by significant interaction between substitution of LA with LO and inclusion of SI on TCH. There were no effects of diet on the plasma contents of HDL-C or LDL-C. Table 4 shows that the abdominal fat percentage was also significantly decreased in birds fed both diets with 4% LO or 4% LO + SI compared to birds fed 4% LA.

### Meat quality

The objective indicators related to meat quality of breast muscle are shown in Table 5. There were no effects of partial or complete replacement of LA with LO, with or without SI, on drip loss, pH, shear values, color attributes, or IMF content of the breast muscle.

**Table 5:** Effects of dietary linseed oil supplementation, with and without soybean isoflavone, on meat quality attributes in the breast muscle of yellow-feathered chickens^1^

Meat quality indicators	4% LA	2% LA + 2% LO	4% LO	2% LA + 2% LO + SI	4% LO + SI	SEM	*P* values			
							Treatment	LO	SI	LO × SI
Color										
Lightness (L*)	57.12	56.32	57.45	56.62	55.12	0.582	0.78	0.90	0.47	0.35
Redness (a*)	14.15	14.55	14.28	13.83	14.95	0.256	0.73	0.51	0.97	0.29
Yellowness (b*)	11.45	10.40	11.00	11.78	10.07	0.335	0.50	0.47	0.88	0.15
pH_24h_	5.71	5.76	5.71	5.70	5.75	0.017	0.80	0.90	0.79	0.23
Drip loss (%)	3.48	3.71	3.79	3.51	3.83	0.115	0.84	0.95	0.35	0.75
Shear force (N)	35.58	38.67	39.40	35.38	36.28	1.075	0.70	0.63	0.41	0.45
IMF (%)	1.113	1.229	1.329	1.263	1.396	0.080	0.79	0.53	0.79	0.93

pH_24h_ = pH 24-h postmortem, IMF = intramuscular fat (as percentage of dry muscle).

1 Data are means for *n* = 6 replicates (2 birds/replicate), dietary treatments were combinations of LA = lard; LO = linseed oil; and SI = 30 mg/kg soybean isoflavone.

2 Treatment, all five diets. LO, dietary LO substituted for half or total LA in the last four diets. SI, dietary linseed oil with or without SI in the last four diets.

### Muscle fatty acids

The fatty acid composition of the total lipids in chicken breast muscle is provided in Table 6. Chickens fed diets containing LO exhibited higher contents (*P* < 0.05) of ALA, EPA, n-3, PUFA and PUFA/SFA, together with lower contents (*P* < 0.05) of palmitate (C16 : 0), arachidonate (C20 : 4n-6), lignoceric acid (C24 : 0), SFA and n-6:n-3 ratio compared with the 4% LA diet. The myristate (C14 : 0), oleate (C18 : 1n-9) and MUFA contents were lower (*P* < 0.05) in chickens fed diets with 4% LO or 4% LO + SI than in those fed the basal diet with LA alone. When 30 mg SI/kg was added to the diets containing LO, the EPA and DHA contents increased further (*P* < 0.05). In the case of EPA, a significant interaction existed between LO level and inclusion of SI.

**Table 6:** Effects of dietary linseed oil supplementation, with and without soybean isoflavone, on fatty acid composition of lipids in the breast muscle of yellow-feathered chickens

Fatty acid(%)	4% LA	2% LA + 2% LO	4% LO	2% LA + 2% LO + SI	4% LO + SI	SEM	*P* values^1^			
							Treatment	LO	SI	LO × SI
C14:0	0.672^a^	0.618^ab^	0.491^c^	0.586^b^	0.492^c^	0.016	0.001	0.001	0.62	0.49
C16:0	25.70^a^	22.53^bc^	21.73^c^	23.89^b^	22.48^bc^	0.332	0.001	0.053	0.064	0.58
C16:1n-7	3.15	3.33	2.53	2.64	2.51	0.128	0.12	0.13	0.24	0.27
C18:0	9.24	8.08	8.38	9.42	9.61	0.205	0.058	0.59	0.010	0.91
C18:1n-9	29.41^a^	29.14^a^	25.64^b^	27.80^ab^	25.87^b^	0.486	0.019	0.012	0.58	0.44
C18:2n-6	20.77	22.27	20.98	20.30	18.96	0.368	0.063	0.12	0.022	0.97
C18:3n-3	0.919^c^	6.93^b^	12.55^a^	5.78^b^	11.47^a^	0.803	0.001	0.001	0.072	0.95
C20:2n-6	0.334	0.282	0.278	0.316	0.306	0.011	0.51	0.79	0.26	0.92
C20:3n-6	0.999	0.660	0.749	0.871	0.845	0.049	0.26	0.77	0.17	0.59
C20:4n-6	5.487^a^	2.998^b^	2.671^b^	3.839^b^	3.459^b^	0.233	0.002	0.48	0.11	0.96
C20:5n-3	0.432^d^	0.818^c^	1.358^b^	1.360^b^	1.697^a^	0.094	0.001	0.001	0.001	0.039
C22:6n-3	0.926^b^	1.049^b^	1.076^b^	1.292^a^	1.317^a^	0.047	0.009	0.48	0.026	0.52
C24:0	1.189^a^	0.530^bc^	0.442^c^	0.749^b^	0.620^bc^	0.059	0.001	0.12	0.095	0.87
SFA	36.80^a^	31.76^c^	31.04^c^	34.48^b^	33.12^bc^	0.504	0.001	0.22	0.011	0.65
MUFA	32.83^a^	32.75^a^	28.41^b^	30.71^ab^	28.54^b^	0.588	0.017	0.014	0.44	0.38
PUFA	29.82^c^	34.77^b^	39.49^a^	33.63^b^	37.78^a^	0.729	0.001	0.001	0.16	0.77
PUFA/SFA	0.811^d^	1.102^b^	1.274^a^	0.975^c^	1.146^b^	0.033	0.001	0.001	0.042	0.65
n-3	2.40^c^	8.68^b^	14.80^a^	8.30^b^	14.35^a^	0.864	0.001	0.001	0.37	0.93
n-6	27.42	26.10	24.68	25.33	23.43	0.453	0.054	0.11	0.32	0.81
n-6/n-3	11.45^a^	3.03^b^	1.68^c^	3.11^b^	1.65^c^	0.687	0.001	0.001	0.87	0.74

LA = lard; LO = linseed oil; SI = 30 mg/kg soybean isoflavone; SFA = saturated fatty acids; MUFA = monounsaturated fatty acids; PUFA = polyunsaturated fatty acids.

1 Treatment, all five diets. LO, dietary LO substituted for half or total LA in the last four diets. SI, dietary linseed oil with or without SI in the last four diets.

a,b,c,d Means marked with different letters in rows differ significantly at *P* < 0.05 (*n* = 6).

**Figure 1:**
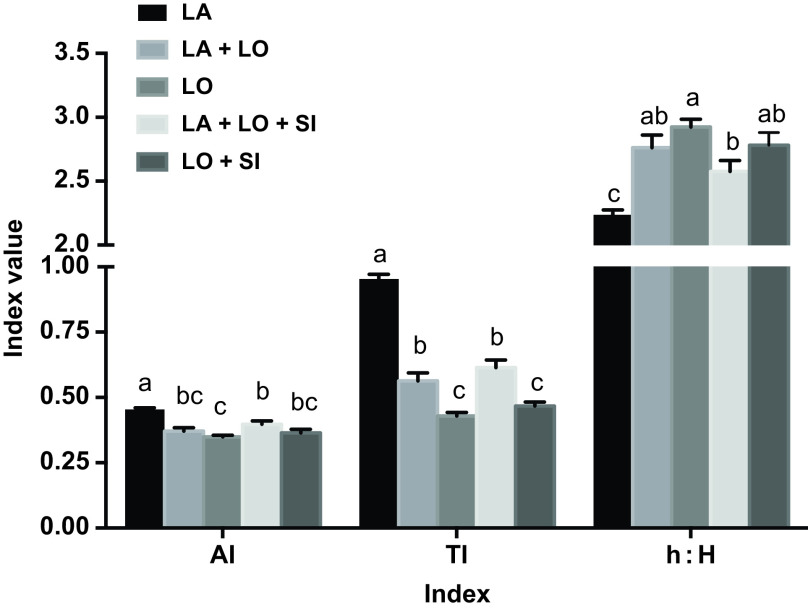
Effects of dietary linseed oil supplementation, with or without soybean isoflavone, on lipid-related health indicators in chicken breast muscle. AI = atherogenic index; TI = thrombogenic index; h : H = hypocholesterolemic/hypercholesterolemic; LA = lard; LO = linseed oil; SI = 30 mg/kg soybean isoflavone. Values with different letters (a to c) within a variable (AI, TI and h : H) indicate significant differences between experimental diets (*P* < 0.05). Error bars indicate standard error of each diet (*n* = 6).

Lipid-related health indicators (AI, TI and h : H) of breast muscle samples were calculated from the fatty acid compositions (Figure 1). The AI and TI indicators showed similar significant decreases with increased inclusion of LO; changes in the h : H index were in the opposite direction. Inclusion of SI had no effect on lipid-related health indicators.

### Muscle expression of genes related to lipid metabolism

As shown in Table 7, the relative transcript abundance of *FADS1*, *FADS2* and *ELOVL5* in breast muscle significantly increased with 4% LO, 2% LA + 2% LO + SI and 4% LO + SI diets compared with the 4% LA diet, and the 2% LA + 2% LO diet being intermediate. Adding SI to the LO diets of birds increased the gene expression of *FADS2* and *ELOVL2* in breast muscle compared to its absence. The transcript abundance of *LPL* increased significantly with LO substitution (2% LA + 2% LO and 4% LO *v*. 4% LA), *HMGCR* by 2% LA + 2% LO and 4% LO + SI, and *CPT1α* by all diets containing LO, with or without SI; there were no significant effects of diet on *FAS* and *SREBP-1* transcripts.

**Table 7:** Effects of dietary linseed oil supplementation, with and without soybean isoflavone, on expression of genes related to lipid metabolism of breast muscle in yellow-feathered chickens

Genes	4% LA	2% LA + 2% LO	4% LO	2% LA + 2% LO + SI	4% LO + SI	SEM	*P* values^1^			
							Treatment	LO	SI	LO × SI
*FADS1*	1.03^b^	1.15^ab^	1.30^a^	1.37^a^	1.30^a^	0.040	0.038	0.16	0.61	0.14
*FADS2*	1.04^c^	1.32^bc^	1.51^b^	1.73^a^	1.79^a^	0.050	0.013	0.12	0.026	0.21
*ELOVL2*	1.06^b^	1.15^b^	1.23^ab^	1.98^a^	2.03^a^	0.111	0.011	0.16	0.053	0.28
*ELOVL5*	1.05^b^	1.41^ab^	1.59^a^	1.62^a^	1.68^a^	0.068	0.015	0.43	0.39	0.47
*FAS*	1.04	0.88	0.98	0.75	0.82	0.039	0.11	0.32	0.067	0.85
*LPL*	1.02^c^	1.40^a^	1.34^ab^	1.10^bc^	1.17^abc^	0.047	0.044	0.94	0.043	0.52
*HMGCR*	1.03^b^	1.29^a^	1.22^ab^	1.11^ab^	1.31^a^	0.035	0.019	0.35	0.60	0.060
*CPT1α*	1.03^b^	2.74^a^	2.18^a^	2.31^a^	2.20^a^	0.158	0.007	0.28	0.55	0.46
*SREBP-1*	1.02	1.28	0.98	1.26	1.28	0.065	0.39	0.50	0.30	0.35

LA = lard; LO = linseed oil; SI = 30 mg/kg soybean isoflavone; *FADS1* = Fatty acid desaturase 1; *FADS2* = Fatty acid desaturase 2; *ELOVL2* = fatty acid elongase 2; *ELOVL5* = fatty acid elongase 5; *FAS* = fatty acid synthase; *LPL* = lipoprotein lipase; *HMGCR* = 3-hydroxy-3-methylglutaryl-coenzyme A reductase; *CPT1α* = carnitine palmitoyltransferase-1α; *SREBP-1* = sterol regulatory element binding protein-1.

1 Treatment, all five diets. LO, dietary LO substituted for half or total LA in the last four diets. SI, dietary linseed oil with or without SI in the last four diets.

a,b,c Means marked with different letters in rows differ significantly at *P* < 0.05 (*n* = 6).

## Discussion

Animal fats and vegetable oils are often used to increase the dietary energy density to meet the requirements of fast-growing chickens. Additionally, there is an increasing demand for functional foods, whereby supplementation with LO (rich in ALA) is becoming an accepted practice to ensure a favorable fatty acid composition of meat. In this experiment with an improved, local breed of chicken, partially or totally replacing dietary LA with LO decreased the ADFI and increased the feed efficiency. These findings agree with the previous work, showing that chickens’ growth performance responds differently to animal or vegetable lipids. For instance, chickens fed diets containing fish oil combined with LO have decreased the feed intake than those fed diets with fish oil combined with LA (Konieczka *et al.*, 2017). Ferrini *et al.* (2008) also reported that broiler chickens fed diets with LO had decreased feed intake and improved feed efficiency compared with those fed tallow. The effect of the type of fat on feed efficiency could be related to the degree of unsaturation, because some authors (Zollitsch *et al.*, 1997; Duran-Montgé *et al.*, 2007) have reported that digestibility of fat increases as the degree of unsaturation increases. It is evident here that substituting 2% or 4% LO for an equivalent amount of LA in the chicken diet improved the feed efficiency. In addition, in the present study, SI had no effect on chickens’ growth performance.

Compared to saturated fats, dietary fats rich in n-3 PUFA increased the susceptibility of chickens to oxidation. In the present study, when the dietary LA was substituted with LO, there were no significant differences in the T-SOD activity, T-AOC, GSH, GSSH and MDA contents in plasma and breast muscle. When 30 mg SI/kg was also added to the diet, the plasma T-SOD activity and muscle GSH contents increased and the MDA content in plasma and breast muscle decreased. A previous study showed that the addition of 40 or 80 mg/kg of SI significantly increased T-AOC and slightly elevated T-SOD activity in the plasma of chickens (Jiang *et al.*, 2007b). Dietary SI at 20 and 40 mg/kg levels enhanced T-SOD activity in chicken meat (Jiang *et al.*, 2014). These findings suggest that supplemental SI would be expected to improve the antioxidative status of chickens.

The present results showed that diets with LO decreased the plasma TG and TCH contents in chickens compared with those fed the diet with LA, with greater decreases when SI was added. This observation is consistent with the finding that diets enriched with n-3 PUFA from LO and fish oil clearly decreased the serum TCH and TG in broiler chickens (Ibrahim *et al.*, 2018). The results explain the decreased abdominal fat found here in chickens given dietary LO. Previous experiments have shown lower abdominal fat deposition in broilers fed dietary LO compared with those fed tallow (Crespo and Esteve-Garcia, 2001; Ferrini *et al.*, 2008).

The current data confirmed earlier work (López-Ferrer *et al.*, 2001; Bou *et al.*, 2005) that dietary LO had no effects on objective indicators of meat quality and IMF content of breast muscle in chickens. Consistent with the earlier work (López-Ferrer *et al.*, 2001), dietary LO had a large impact on fatty acid composition in chicken breast muscle in the present study, resulting in more n-3 PUFA (ALA, EPA, and DHA) with less SFA and MUFA. The interesting additional finding here was that adding SI to the LO diet further increased the deposition of EPA and DHA in breast muscle, presumably because of the antioxidant property of the isoflavone. This interpretation is consistent with the effects of other antioxidant additives; Pappas *et al.* (2012) found that breast muscle contents of long-chain PUFA (C20:3n-6, C20:4n-6, EPA, DHA and docosapentaenoic acid (22:5n-3; DPA)) increased linearly with dietary inclusion of selenium (0.15, 0.3 and 3 mg/kg) in chickens. Haug *et al.* (2007) similarly found that high dietary selenium increased the contents of the long-chain n-3 PUFA (EPA, DPA and DHA) in broiler meat from the intake of plant oils containing n-3 PUFA.

The present study has also found that dietary LO substituted for half or all of the LA significantly reduced AI and TI and increased h : H in the chicken meat. Accordingly, a lower AI and higher h : H ratio and n-3 content of meat from LO-supplemented chickens compared with LA have been recently reported (Milankovic *et al.*, 2019). Lower values of AI and TI indicate a healthier ratio with regard to fatty acids that reduce platelet aggregation and the potential for coronary diseases; conversely, a higher h : H fatty acid ratio indicates a more nutritionally suitable fatty acid profile (Omri *et al.*, 2019). These results indicate that dietary supplementation with LO plus SI has a positive effect on the nutritional value of chicken meat, expected to be beneficial for human health from its increased content of n-3 PUFA.

Chicken meat has the potential to be a sustainable source of ALA, EPA and DHA as chickens are capable of synthesizing these longer n-3 PUFA from dietary-derived ALA (Gregory and James, 2014). In the present study, the muscle expression of several relevant genes (*FADS1*, *FADS2*, *ELOVL2* and *ELOVL5*) was generally increased by dietary LO with SI supplementation in the birds. To a certain extent, these findings were similar to that found earlier. Jing *et al.* (2013) found that hepatic gene expression of desaturase and elongase (*FADS1*, *FADS2*, *ELOVL2* and *ELOVL5*) increased as the dietary ratio of 18 : 2n-6 (LA)/ALA progressively declined; expression was highest in birds fed lower LA/ALA diets. The present results indicate that intake of dietary LO with SI in chickens resulted in the synthesis of n-3 PUFA in breast muscle by regulating the expression of these relevant genes.

In conclusion, dietary replacement of half or all LA with LO increased feed efficiency and markedly enriched the meat of chickens with n-3 PUFA (ALA, EPA and DHA); it also notably improved lipid-related health indicators (lower AI, TI and higher h : H ratio) and decreased lipid-related indicators (plasma TG, TCH; abdominal fat percentage) without adverse effect on the meat quality. Adding the antioxidant SI to the LO diets further increased the muscle contents of EPA and DHA, decreased the plasma TG and TCH levels and improved the antioxidative status of chickens. Interactions were detected between substitution of LA with LO and inclusion of SI on EPA and TCH contents. Inclusion of LO with SI in the chicken’s diet increased the muscle expression of genes related to lipid metabolism, viz., *FADS1*, *FADS2*, *ELOVL2* and *ELOVL5*. These results indicate that diets supplemented with LO can effectively improve the nutritional value of chicken meat, and this was even better when LO was given together with SI.

## Software and data repository resources

The authors declare that the data of this research are not deposited in any official repository.

## Supplementary material

For supplementary material accompanying this paper visit https://doi.org/10.1017/S1751731120001020. click here to view supplementary material
