# Supplementary material for: Effects of dietary incorporation of linseed oil with soybean isoflavone on fatty acid profiles and lipid metabolism-related gene expression in breast muscle of chickens
Source: Animal. 2020 May 19;14(11):2414–22. doi: 10.1017/S1751731120001020 (PMC7538340; doi:10.1017/S1751731120001020)
Supplement: Supplementary file 1 [file S1751731120001020sup001.docx]

**Effects of dietary incorporation of linseed oil with soybean isoflavone on fatty acid profiles and lipid metabolism-related genes expression in breast muscle of chickens**

Z.Y. Gou^#^, X.Y. Cui^#^, L. Li, Q.L. Fan, X.J. Lin, Y.B. Wang, Z.Y. Jiang, S.Q. Jiang^*^

*Animal*

**Table S1** *Primer sequences of Yellow-feathered chickens used for quantitative real-time PCR^*^*

| Genes | Primer sequence (5'-3') | Accession No. | Amplicon (bp) | Ta (°C) |
| --- | --- | --- | --- | --- |
| *FADS1* | F: CTTGGCGAACAAAAGAAGAAAT  R: CCCAGTAAGGGCAGGTAGGT | XM_421052.5 | 200 | 60 |
| *FADS2* | F: CTGAGGAAGACAGCAGAGGACAT  R: GCAGGCAAGGATTAGAGTTGTG | NM_001160428.2 | 153 | 60 |
| *ELOVL2* | F: TTTGGCTGCCTCATGTTCCA  R: TGTGACGGGGGTTTCCTTTG | NM_001197308.1 | 123 | 60 |
| *ELOVL5* | F: ATTGGGTGCCTTGTGGTCA  R: AGCTGGTCTGGAAGATTGTCA | NM_001199197.1 | 180 | 60 |
| *FAS* | F: GGTCAGTGCTGCACGAAAT  R: CATCTCATACACTCGTCCAAATC | NM_001199487.1 | 137 | 58 |
| *LPL* | F: GGTGACCTGCTTATGCTA  R: ATATTGCTGCCTCTTCTC | NM_205282.1 | 187 | 58 |
| *HMGCR* | F: TGTTGTAAGGCTGCCCTCTG  R: TAGGCGGGCAAACCTACTTG | NM_204485.2 | 118 | 60 |
| *CPT1α* | F: AGGACCAAGGCTTCAGTGGT  R: GGTCCACGCCCTCTAAGGTA | NM_001012898.1 | 102 | 60 |
| *SREBP-1* | F: GCCCTCTGTGCCTTTGTCTTC  R: ACTCAGCCATGATGCTTCTTC | AY029224 | 130 | 60 |

^*^Ta: annealing temperature; *FADS1*: fatty acid desaturase 1; *FADS2*: fatty acid desaturase 2; *ELOVL2*: fatty acid elongase 2; *ELOVL5*: fatty acid elongase 5; *FAS*: fatty acid synthase; *LPL*: lipoprotein lipase; *HMGCR*: 3-hydroxy-3-methylglutaryl-coenzyme A reductase; *CPT1α*: carnitine palmitoyltransferase-1α; *SREBP-1*: sterol regulatory element binding protein-1.
